# Development and effectiveness analysis of a safety management model for SMEs integrating lean management innovation with SQCDP framework

**DOI:** 10.1371/journal.pone.0316299

**Published:** 2025-01-13

**Authors:** Yichen Yin, Xinyi Ouyang, Jinggang Zhang, Haoyu Chen, Haochen Xu, Wanting Zhang, Chongqin Lin, Zulin Zhou

**Affiliations:** 1 School of Safety Engineering of North China Institute of Science and Technology, Dongyanjiao, Beijing, People’s Republic of China; 2 Department of Electrical and Computer Engineering of McGill University, Montreal, Quebec, Canada; 3 Wenzhou Lucheng Emergency Bureau, Wenzhou, Zhejiang, People’s Republic of China; 4 School of Mining Safety of North China Institute of Science and Technology, Dongyanjiao, Beijing, People’s Republic of China; King Khalid University, SAUDI ARABIA

## Abstract

This study organically integrates the safety, quality, cost, delivery, and people (SQCDP) management mode and lean management to create the SQCDP + lean safety management (SLSM) mode, which addresses certain problems faced by China’s small- and medium-sized enterprises (SMEs), such as imperfect safety management systems, poor regulatory implementation, challenging problem correction, and perfunctory management. It then explains the benefits proposed mode for SMEs and the establishment of a novel mode that aligns with the current safety production and operation management of SMEs. The application of the SLSM mode to a private machinery manufacturing company in Wenzhou resulted in the effective shouldering of safety responsibilities by the company, a year-on-year decrease in accident rates, and a significant increase in production efficiency, thereby providing corporate managers with guidance and suggestions for making improvements.

## 1. Introduction

This year (2023), The Chinese Ministry of Industry and Information Technology (MIIT) aims to increase the number of specialized, innovative SMEs nationwide beyond 80,000. SMEs, as critical components of the national economy, play an essential role in promoting economic diversification, reducing employment pressures, and enhancing market dynamism [1]. However, as they develop further, SMEs face increasingly pronounced safety issues, with frequent accidents and severe occupational hazards significantly impacting their growth and economic performance. Research on SME safety management is currently limited [2]. In 2024, Yang Daojian and his team introduced the "Context-Process" framework, constructing a theoretical model to explore work alienation mechanisms among SME safety managers and analyzing its influencing factors [3]. Although this study investigates employee alienation within SME safety management and proposes solutions, it does not fully address the comprehensive safety management needs of SME administrators. In 2022, Li Zheng conducted a study on prevalent safety issues in dust explosion-prone SMEs, recommending improvements through empirical, case, and comparative analyses [4]. However, the study’s scope is narrow, and it lacks detailed quantitative analysis, focusing solely on a specific SME type. In 2020, Wang Peng introduced a "collaborative" SME safety management model based on the SCORE methodology [5]. However, this model lacks a clear, culturally adapted approach for China and does not adequately address local adaptations post-introduction. This research, confined to regional pilot projects, lacks sufficient theoretical depth and clear guidelines for SME implementation, leading to credibility issues from inadequate statistical analysis. In 2006, Lu Yumei and colleagues conducted an extensive study on the current state of SME safety management, proposing strategies to enhance employee safety awareness and develop market entry and exit mechanisms [6]. Despite its comprehensive nature, this research does not provide a readily adoptable model for SMEs and has become somewhat outdated for the current safety management context in China. Also in 2006, Li Chuangui proposed the "4+1" safety management and supervision model from an external regulatory perspective, offering a clear theoretical framework [7]. However, this model primarily focuses on adjusting SME safety management through external factors, failing to enhance internal productivity or foster a proactive safety and innovation culture, leading to passive business compliance. In 2017, Chu Aiguo recommended integrating the SQCDP model to strengthen foundational management, enhance management efficiency, and reduce operational costs and communication barriers within enterprises [8]. However, the integration of certain theories in the SQCDP model appears forced, offering limited applicability and requiring significant initial investment, which may not align well with practical production or fully meet Chinese conditions. In 2005, Li Yongxiang drew inspiration from Japan’s Toyota to propose a "Lean Management" model for China, blending advanced management concepts with lean production techniques [9]. However, the outdated data and complex structure in his study render it unsuitable for SMEs.

The studies [1–9] discussed often suffer from a lack of foundational theory, simplistic modeling, disconnection from practical applications, and inadequate guiding principles. These shortcomings impede the effective resolution of conflicts between safety management and economic benefits in Chinese SMEs. Studies have indicated that small and medium-sized enterprises (SMEs) face significant challenges in safety management. These challenges include a lack of targeted scientific guidance, inadequate risk control mechanisms, superficial hazard identification, and insufficient coordination between production stages [10, 11]. To address these issues, this paper employs the SQCDP management model to provide SMEs with key multidimensional information, identify primary challenges, and offer precise directions for production management reform. The model integrates lean management concepts, automation transformation, a dual prevention mechanism, and a government-industry-university-research collaboration model. This integration results in the innovative "SQCDP + Lean" safety management model (SLSM). The model aims to address the identified issues while optimizing SMEs’ financial chains, enhancing production process efficiency, and improving market competitiveness. Ultimately, it seeks to provide guidance and insight for SME development and transformation.

## 2 Overview of SQCDP visual management and lean management models

### 2.1. Overview of SQCDP visual management

SQCDP visual management refers to the visualization of the key information in a production setting (safety, quality, cost, delivery, and people) to achieve production objectives. It helps managers and workers to identify, classify, analyze, track, and solve problems, thereby reducing the related economic losses, casualties, and risks. SQCDP is an advanced management mode that can maximize the efficiency and benefits of a team and its workshop [12].

### 2.2 Lean management in corporate safety management mode

Lean management focuses on human behavioral control and aims to improve the performance of workers by optimizing their work processes. It deploys multiple management means and advantages to enhance customer satisfaction and product quality, eliminate nonessential costs, accelerate processes, and radically transform capital structures to maximize the benefits of enterprises using the resources at hand [13]. It is crucial to limit the exclusive focus on high production efficiency and instead emphasize customer service. Furthermore, improving resource utilization in production and operations can enhance overall enterprise performance. Enterprises must also balance efficiency improvements with risk control, applying lean tools to identify processes that are both high-efficiency and low-risk. Although many industries have experimented with lean safety management to varying degrees, such experiments are mostly in the exploratory stage and confined to management approaches. Moreover, considering the generalization of the lean management concept and the individualization of efficient lean management [12], SMEs, which are highly vulnerable and in need of lean management, are suffering from a shortage of sophisticated management concepts, modes, or methods. They also lack precise, targeted corrective measures that are linked with and can be applied to related industries and enterprises [14].

## 3 SME safety management problems

### 3.1 Poorly constructed safety management systems

Due to their abundance in number, limited size, diversity, and private ownership, SMEs encounter problems such as centralized management, subjective decision-making, insufficient attention to production safety, and excessive emphasis on production efficiency by management. Thus, when contradictions between safety and production need to be addressed urgently, SME managers tend to prioritize the latter. Moreover, SMEs struggle with insufficient accident experience summaries, inadequate safety skills training for personnel, insufficient internal safety education and training, low employee education levels, and a lack of well-established operational processes. In environments where production is prioritized over safety, such shortcomings increase the likelihood of accidents.

Current safety management modes and regulations are primarily designed for medium and large enterprises. However, the difference between SMEs and large enterprises in management systems, talent allocation, and capital is so significant that SMEs are extremely lacking and unsystematic in safety management and therefore are not covered by relevant safety production regulations [15]. Worse still, the scarcity of safety management results in challenges in the upgrading and advancement of safety production technologies already subject to considerable restrictions, thus negatively affecting the production safety and long-term production benefits of SMEs, such as by introducing hidden hazards.

### 3.2 Limited coverage of safety management

For many SMEs in China, safety management is a method that is primarily based on practical experience rather than a systematic approach that combines such experience with scientific methods. Thus, SMEs often lack comprehensive safety production management systems that take into account production processes, technologies, quality control, and safety management modes. This can lead to difficulties in identifying specific weaknesses and unknown safety risks in situations where existing experience fails, or new equipment is introduced. Consequently, SMEs struggle with safety production management problems that need to be resolved immediately, such as outdated technology and production equipment, hidden equipment maintenance risks, poor adaptability to new equipment, and ineffective human—machine safety systems [16].

### 3.3. Safety management practices in small and medium-sized enterprises (SMEs) are outdated

In China, some small and medium-sized enterprises (SMEs) prioritize high efficiency and automation, focusing on short-term gains rather than long-term sustainability. This approach often results in a neglect of product and service quality, limiting growth and reducing market competitiveness. Customer feedback and service are undervalued, leading to inadequate understanding of customer needs and ineffective after-sales support. This not only results in customer loss but also prevents the use of customer feedback for product improvement and innovation. The lack of a comprehensive reform mindset, encompassing product value, customer service, and resource allocation, is a fundamental cause of imbalanced costs and revenues. Ultimately, outdated safety management practices among SMEs hinder adaptation to rapid market changes, significantly restricting high-speed and high-quality development [17–20].

## 4 Integration of SQCDP with lean management in SME safety management (SLSM mode)

### 4.1 Necessity and paths of implementing SLSM mode in SMEs

The market competitiveness of enterprises is significantly influenced by their level of management. Numerous SMEs encounter challenges due to limited economic momentum, compromised product reliability, and inadequate safety management. Adaptive, practical safety management not only accelerates economic momentum and optimizes the capital chain but also promotes the rationalization and enhancement of production systems, thereby elevating the quality and reputation of enterprise products. While the SQCDP management mode offers guidance for addressing practical business challenges, it does not constitute a long-term strategy capable of effectively supporting the sustained, cyclical development and closed-loop processes of enterprises [16, 21]. This limitation stems from a lack of human-centric management methodologies and concepts that could be effectively augmented by lean management practices [21].

The advancement of SMEs hinges on addressing two fundamental challenges: enhancing and optimizing complex production and management systems, and reconciling the contradictions between economic benefits and safety management [22]. Furthermore, the aforementioned problems are inherently interconnected. In the current business climate, SMEs must increase their revenue and reduce costs to sustain their regular internal operations. An SME can seamlessly integrate SQCDP with lean management to develop the SLSM model, harnessing SLSM to systematically optimize the enterprise. This includes implementing real-time monitoring, emphasizing critical production factors, adopting scientific management practices to refine management and production processes, enhancing workshop production line planning and layout, and strengthening process quality management and control, thereby establishing a novel safety management paradigm tailored to its domestic market conditions and scientific management philosophy.

### 4.2 Content of the SLSM mode

The SLSM mode is divided into four major modules corresponding to the five dimensions of the SQCDP management mode. Lean safety (S), lean quality (Q), lean cost (C), and lean people management (P) are the modules that are specifically connected through SQCDP and make lean management the production concept of SMEs. The lean delivery (D) dimension is decomposed across the other modules.

#### 4.2.1. Lean safety module

Safety is essential for cooperative production and operation activities. Given today’s fierce market competition and shortened upgrading cycles of production equipment, enterprises must rely on their core competencies to survive, such as establishing risk barriers, building safe production systems with lean safety at the core, and adopting sustainable development strategies [23]. In light of this, SMEs can build lean safety production management systems via a three-step approach: one standardization, five improvements, and two introductions.

**Fig 1:**
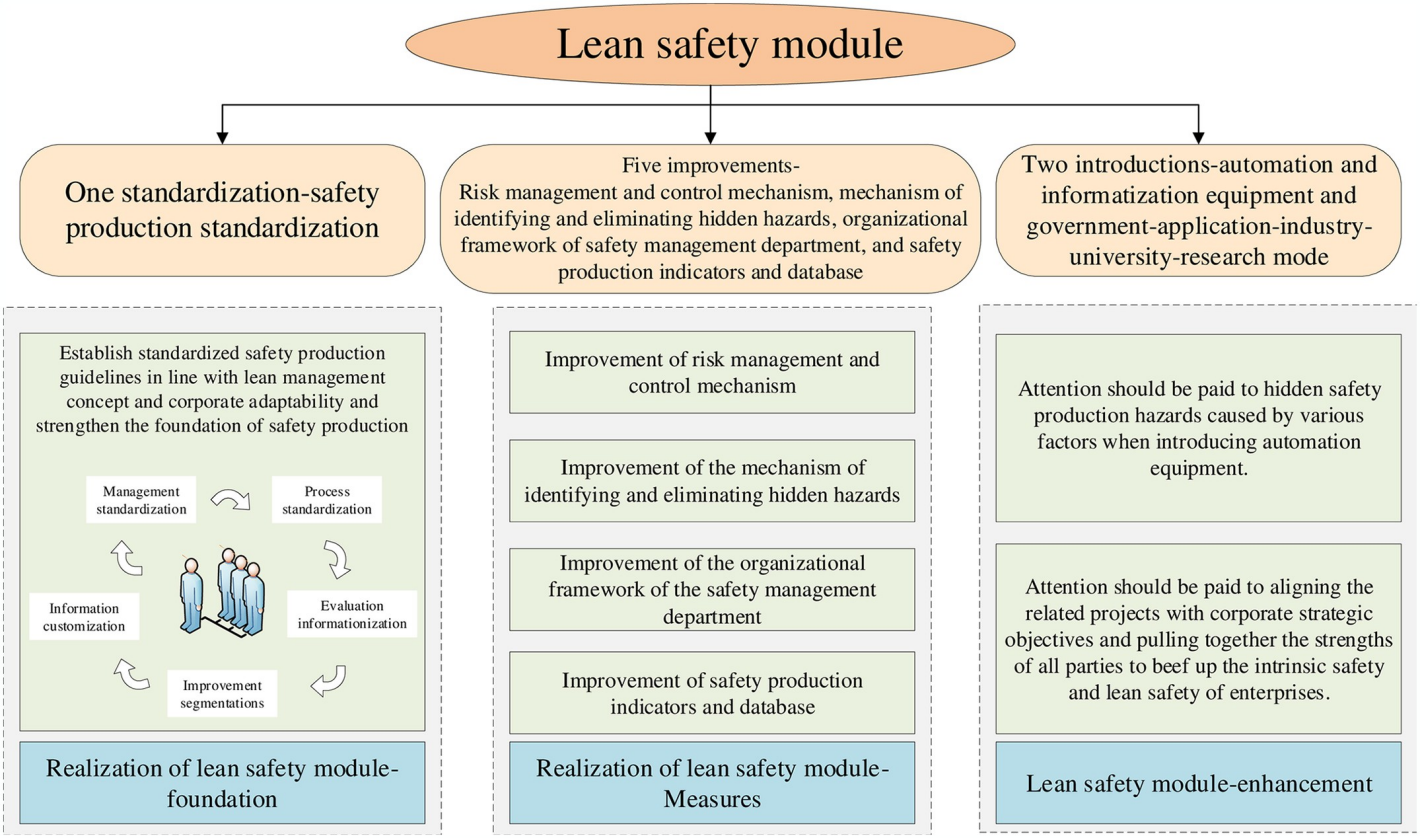
Theoretical framework of the lean safety module.

Standardization in the context of industrial safety refers to the systematic regulation of production safety protocols. This paradigm encompasses five key enhancements: the refinement of risk management and control systems, the development of methodologies for identifying and mitigating latent hazards, the structuring of organizational frameworks within safety management departments, and the enhancement of safety production metrics and databases. Furthermore, the integration involves two critical introductions: the deployment of automation and informatization technologies, and the adoption of a collaborative model involving government, application, industry, university, and research entities. This tripartite approach initiates with standardization, which solidifies foundational safety production tasks and achieves alignment with lean management principles and corporate adaptability. This initial phase is segmented into five components: management standardization, process standardization, the informatization of evaluation, segmentation of improvements, and customization of data. These five enhancements not only facilitate the effective execution of the dual-prevention mechanism but also provide crucial data on risk, incidents, training, occupational health, and the scope and nature of concealed hazards. Additionally, these improvements support auxiliary functions in safety production, enhance conditions for safety operations, and elevate safety management departments by an incremental hierarchical level, thereby enabling organizations to maintain a balance between production and safety within their structural framework. The subsequent introduction of automation technologies and the collaborative government-application-industry-university-research model is pivotal in circumventing hidden safety risks, potentially overlooked during the automation integration due to inadequate assessment of industrial software’s safety prevention capabilities. Hence, the design of automation equipment must incorporate stringent safety standards as compulsory criteria for equipment suppliers. In deploying the collaborative model, the objectives for enhancement should be strategically aligned with corporate goals. This strategy not only fortifies the synergy between industry, academia, and research entities but also maximizes the utility of open, innovative governmental platforms and pertinent policies. Ultimately, this facilitates a holistic enhancement of lean safety practices by amalgamating the strengths of all involved stakeholders. The detailed structural framework and logical relationship of this module are shown in Fig 1.

#### 4.2.2. Lean quality module

**Fig 2:**
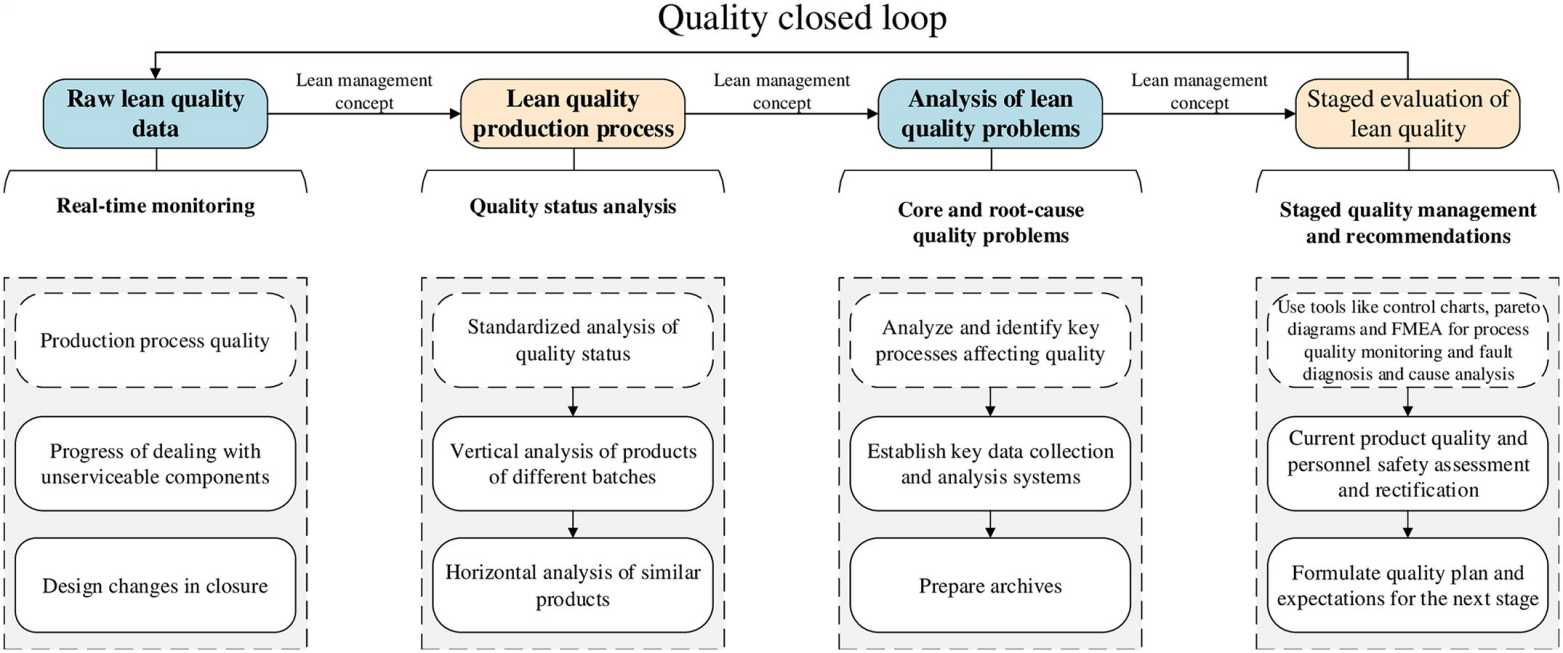
Prevention and self-correction theoretical framework of the lean quality module.

The lean quality framework can be succinctly described as encompassing four processes and a singular closed-loop system. Real-time monitoring of raw quality data—including aspects such as production process quality, the management of unserviceable components, and design modifications at closure—is essential. Moreover, a standardized vertical analysis of the production process’s quality status should be systematically executed. In conjunction with a horizontal analysis of analogous products, this rigorous monitoring and analysis framework will enable enterprises to formulate strategic recommendations aimed at managing production process quality, refining the production workflow, and achieving precise production technology goals and quality inspection benchmarks in subsequent phases. Consequently, enterprises will achieve effective oversight of the singular quality dimension and establish a scientifically robust closed-loop system for self-innovation, as illustrated in Fig 2.

During the integration of lean quality and safety modules, it is essential to provide root-cause solutions that address the core of identified issues [24]. For example, in critical processes such as injection molding, torque testing, and seal testing, establishing data collection and analysis systems for key raw data, along with preparing comprehensive production process archives, is an effective method for controlling process quality. These systems not only guarantee that products adhere to quality standards but also ensure their safe transportation from warehouses and distribution. Under specific conditions, the application of analytical tools such as control charts, Pareto diagrams, and Failure Mode and Effects Analysis (FMEA) enables the execution of process quality monitoring, fault diagnosis, and causality analysis. This approach is crucial in preempting safety risks to customers or workers stemming from product or equipment malfunctions.

#### 4.2.3. Lean cost module

**Fig 3:**
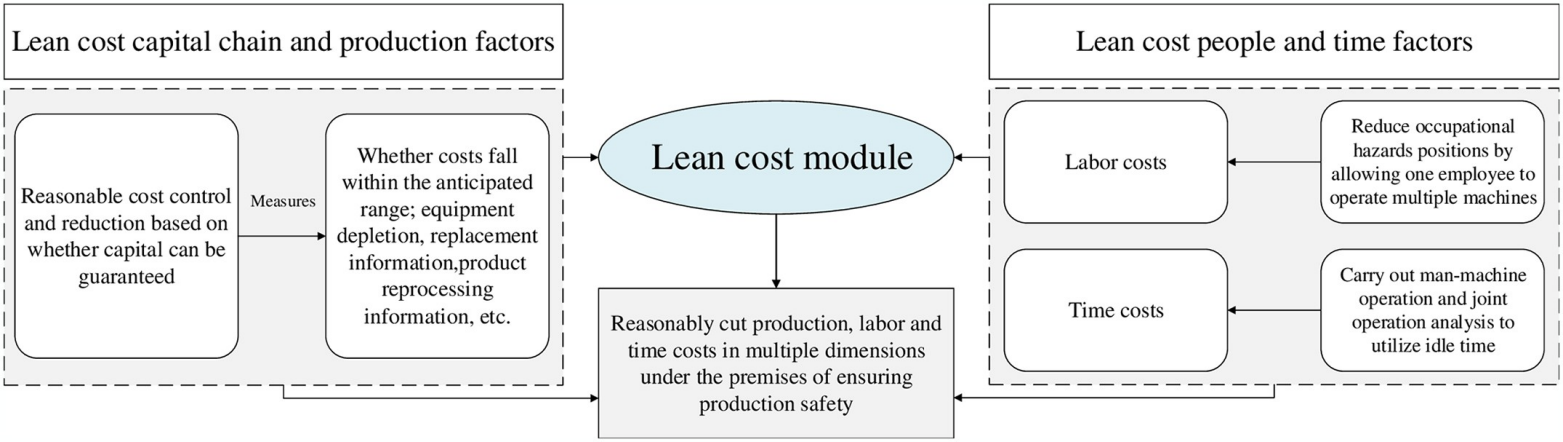
Theoretical framework of the lean cost module.

The lean cost module secures effective cost control and reduction through the deployment of two stringent evaluation criteria: the alignment of the production process with predefined expectations, and the thorough resolution of any financial discrepancies within the production process. This criterion encapsulates factors such as equipment depreciation, detailed replacement logistics, product reprocessing insights, and the consistency of all incurred costs with projected financial boundaries. Besides the collection and analysis of production data, it is imperative for enterprises to acquire quality assurance data, which is vital for the robust assessment and analysis of product field reliability and the associated costs of quality assurance. Consequently, this approach will significantly enhance product reliability, optimize quality assurance strategies, and substantially reduce costs associated with quality assurance services, as illustrated in Fig 3.

In SME workshops, one person can operate multiple machines in certain areas, leading to reduced labor costs and exposure to occupational hazards. Nevertheless, workers have idle moments, indicating potential room for enhancing efficiency [25]. However, improving work efficiency may bring new safety risks, such as worker fatigue. To optimize time utilization safely while ensuring safety, enterprises can perform human—machine operation and joint operation analysis to take advantage of idle times.

#### 4.2.4. Lean people management module

**Fig 4:**
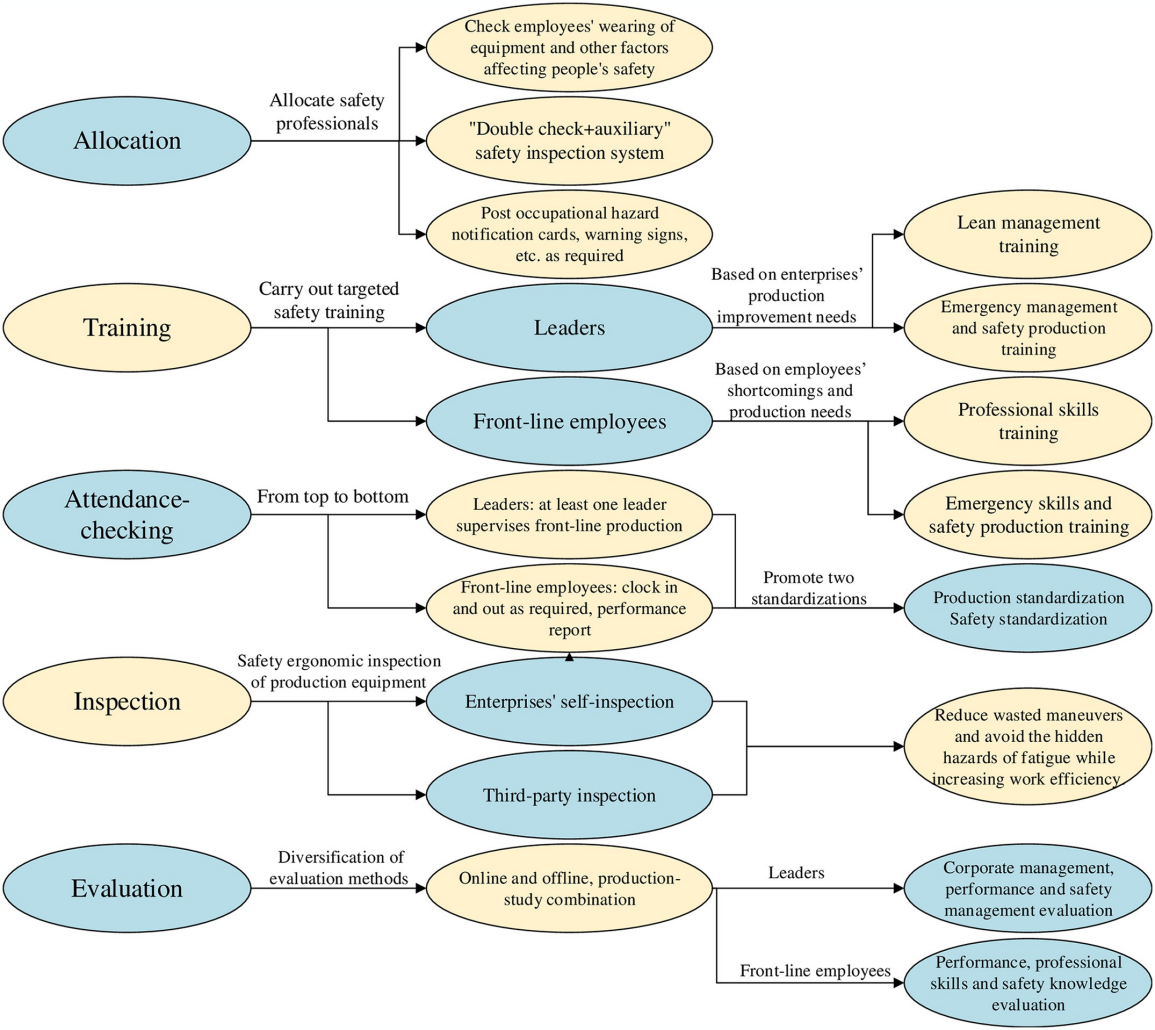
Theoretical framework of the lean people management module.

People orientation is at the core of the effective implementation of the SLSM mode. SMEs have limited head counts and generally low employee educational levels [26]. Therefore, for the lean people management module of SMEs, the key is to reduce labor costs and improve operational efficiency while ensuring the safety of people and machines. This model can be achieved through five aspects: allocation, training, attendance checking, inspection, and evaluation. Its specific framework is shown in Fig 4.

Allocation entails deploying full-time safety professionals to workshops, where they conduct comprehensive pre-operational checks on numerous factors including equipment operation, occupational disease risks, adherence to personal protective equipment protocols, and the physical and psychological well-being of employees, as well as certification requirements for specific roles, before green-lighting production. Worker safety can be optimized through an exhaustive, specialized, and lean inspection across various job roles. This is achieved by implementing a ’double check + auxiliary safety inspection system’, which involves rigorous safety evaluations conducted by safety professionals, individuals accountable for each position, and additional spot checks by on-duty leaders.

Training programs are specifically designed for both frontline employees and corporate leaders. For frontline employees, ongoing training sessions, comprehensive guidance, and strict codes of conduct are implemented to enhance safety awareness. Additionally, three-tier routine meetings are held to address any discrepancies and unsafe practices observed in daily operations, ensuring that employees are fully cognizant of job-related risks and are prepared with appropriate emergency responses. For enterprise leaders, specialized safety management training tailored to production enhancement needs is essential, aimed at elucidating the pivotal role of safety management in bolstering production and thereby ensuring the robust operation of the enterprise.

Attendance checking requires the strict checking of personnel attendance and the establishment of a top-down attendance management system. For production standardization and safety standardization, safety production regulations must be followed during production, starting with leadership. Moreover, personal performance reports must be formulated by combining work efficiency and employees’ clocking in and out at specified locations, among others [27].

The inspection involves the safety and ergonomics inspection of displays, worktable heights, and the design of other equipment and the self- and third-party inspection of equipment. Employees’ physiological and psychological characteristics should be considered to reduce wasted maneuvers (e.g., workbench or other equipment that are not consistent with safety ergonomics) and avoid the hidden hazards of fatigue while increasing work efficiency.

The performance, professional skills, and safety knowledge of different groups of people are regularly assessed. For enterprise leaders, the corporate management mode should also be evaluated. Moreover, online and offline combined production—study modes can be utilized. For daily production links prone to accidents, adjustments should be made promptly based on multidimensional data statistics and feedback [28]. This establishes the foundation for improving the overall work quality of employees and constructing a corporate culture.

#### 4.2.5. Summary of continuity, mutuality, and implementation process between SLSM modules

**Fig 5:**
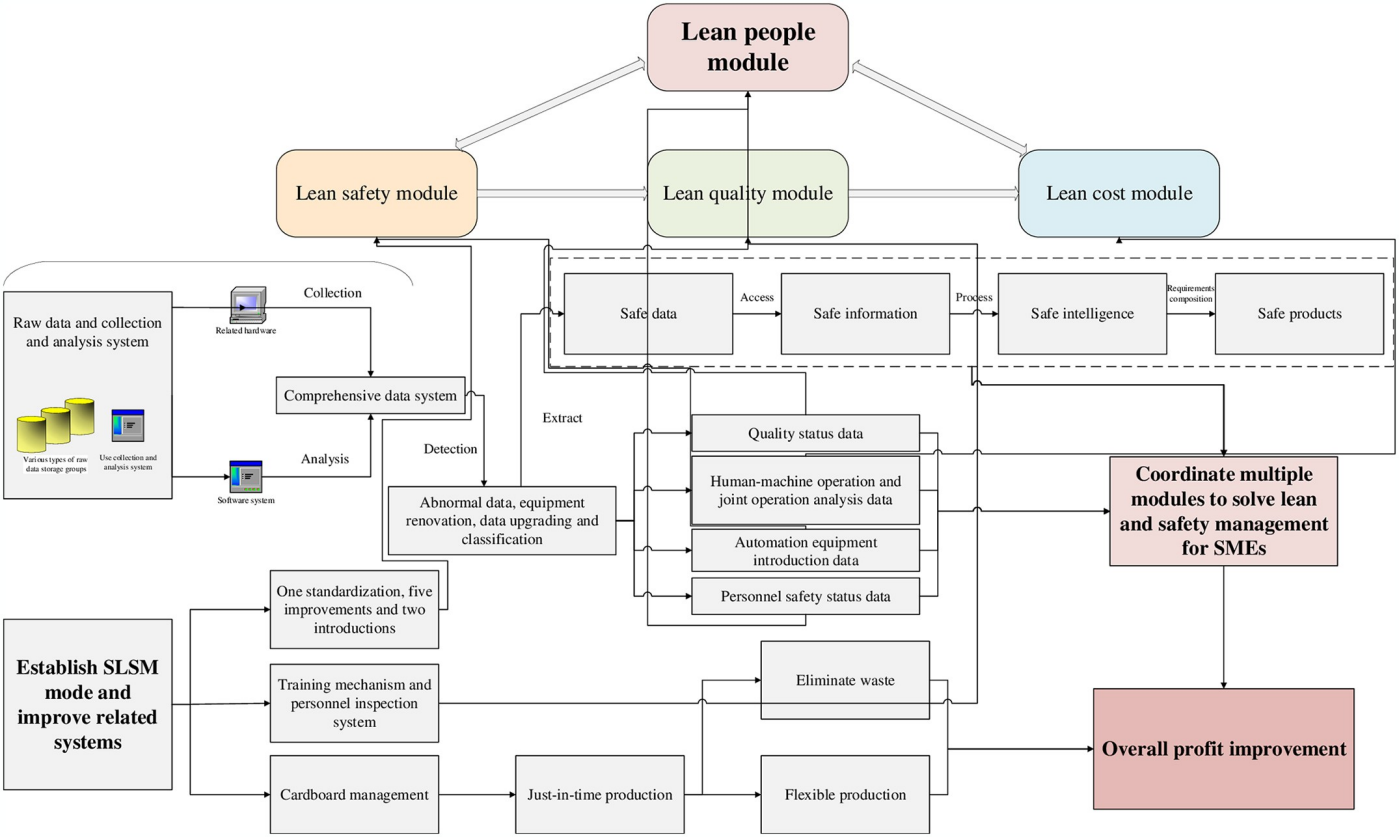
Operation framework of the SLSM mode.

**Fig 6:**
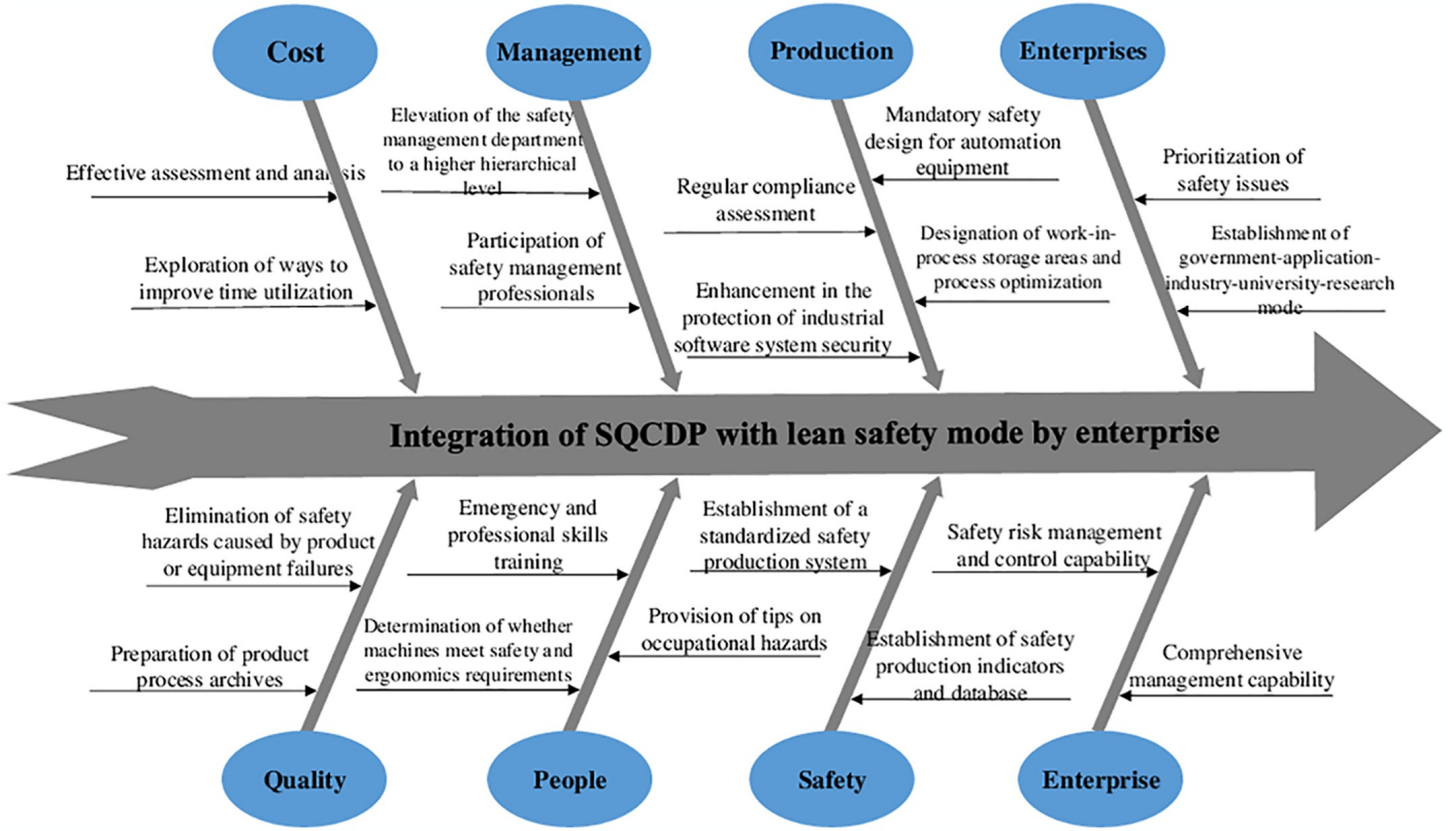
SLSM fishbone diagram.

Each component of the proposed safety management model, which integrates SQCDP with lean management principles, plays a pivotal role in the robust development of SMEs. The continuity and interdependence between these modules mirror the cohesive production logic inherent in the five dimensions of SQCDP. In the SLSM framework, the lean safety module serves as the foundational operating base, while the lean people management module constitutes the core, a relationship corroborated by both the lean quality and lean cost modules. The lean safety module catalyzes internal structural reforms within production processes, thereby further enhancing product quality and reducing costs. The lean safety module drives internal structural reforms in production processes, enhancing product quality and reducing costs. Activities such as allocation, training, attendance monitoring, inspection, and evaluation within the lean people management module strengthen lean management practices and foster corporate culture among staff, ensuring smooth operation and self-regulation of other modules, as shown in Fig 5 [29–31]. The implementation of the SLSM mode initiates with the 5S method, serving as both a concrete strategy and a framework for application, and culminates in the thorough integration of lean management concepts throughout each production step. A closed-loop system is established by continuously identifying issues, implementing improvements, reducing waste, cutting costs, boosting production flexibility, elevating product market competitiveness, augmenting enterprise profitability, developing human resources, and securing guarantees through targeted internal training, as illustrated in Figs 5 and 6.

## 5 Practical application and effectiveness of SLSM mode

### 5.1 Profile of the implementing company

Wenzhou Runxin Manufacturing Machine Co., Ltd was founded in 2000. Runxin was titled with National High-tech Enterprise, National Specialized, Fined, Peculiar and Innovative “Little Giant” Enterprise, Zhejiang Hidden Champion Enterprise, Zhejiang Patent Model Enterprise, Zhejiang Hidden Champion Enterprise, Zhejiang Patent Model Enterprise, Wenzhou First Batch of “The Most Beautiful Factories”, Wenzhou Leading Enterprise and Wenzhou High Integrity Enterprise, has more than 60 invention patents and utility model patents, and has passed CE an RoHs certifications.

Runxin is primarily engaged in multi-functional flow control valve for water treatment systems, domestic water softeners, ceramic hard sealing ball valve and control valve for solar water heater. The main manufacturing technologies include injection processing, NC processing, ultrasonic welding and hot plate welding. The company currently has 482 staffs, including 2 security officers, 10 special operation officers.

### 5.2 Practical application and automation transformation of SLSM model

**Fig 7:**
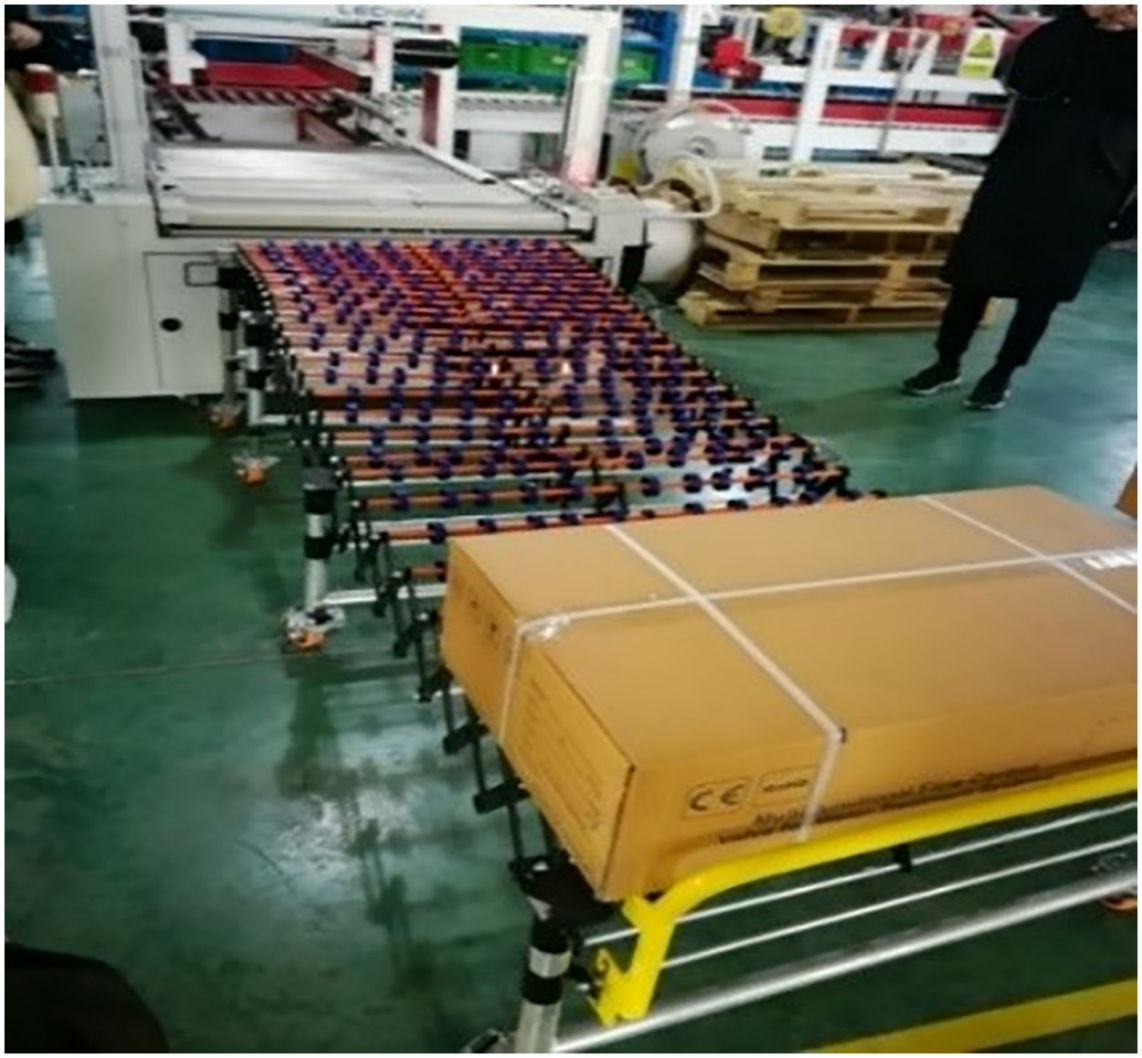
Current modular production of Wenzhou-based machinery enterprise after implementing SLSM mode (1).

**Fig 8:**
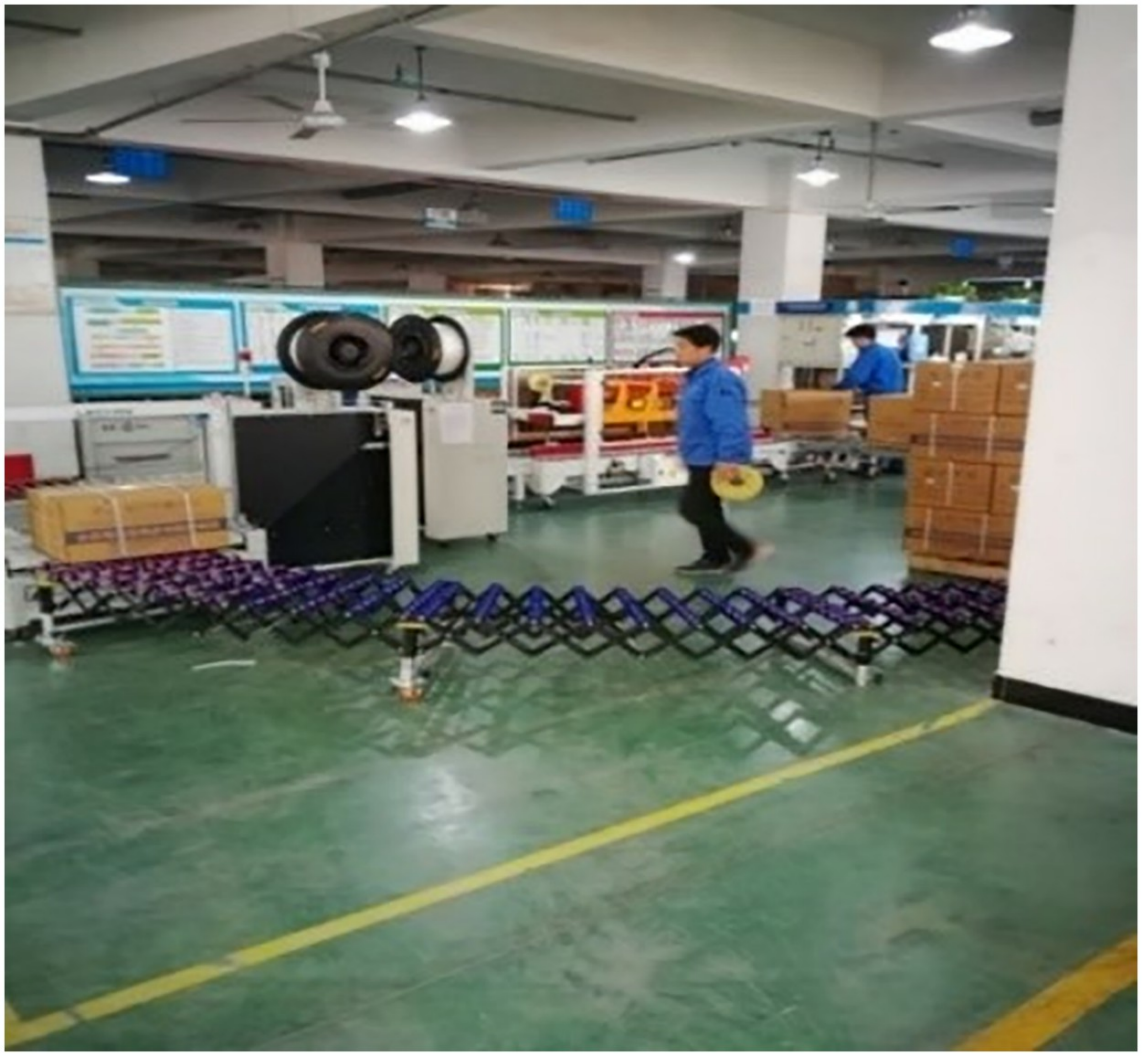
Current modular production of Wenzhou-based machinery enterprise after implementing SLSM mode (2).

Runxin has adopted the SLSM mode since September 2019. During the period, the company set up research development centers, testing centers, water quality testing departments and measurement departments and so on. Meanwhile, Runxin has significantly increased its investment in automation projects and has integrated safety considerations into automation transformation using the lean safety concept as the guiding principle. It has also executed new transformation and expansion projects, created a “safety risk identification, assessment, management, and control” table, and integrated the SQCDP dimensions. Measures including automatic personnel reduction and replacement of people with robots have lowered the operational risks of employees, thereby achieving lean safety and intrinsic safety. Figs 7 and 8 shows the transformation measures undertaken by the enterprise after implementing the SLSM mode. Additionally, the enterprise has promoted the idea of “relying on professionals to deliver the best results.” It has built platform for government, application, education, research and application, invite the government, associations, universities, enterprises and other units to form an expert team as security consultants to assist enterprises in planning and give technical support.

The enterprise’s specific transformation measures that merge SLSM mode and safety human- machinery engineering is partially shown in Table 1.

**Table 1:** Transformation measures of automation and safety ergonomics integrating SLSM mode.

	Transformation measure	Efficiency improvement	Removed personnel
**1**	Modified torque automatic assembly equipment requiring two employees operating control valves; streamlined three work processes into one automation equipment	Decreased the accumulative standard working time of the original unit (68 s) to 25 s (63% increase in efficiency)	**2**
**2**	Introduced automatic packaging lines to workshops of electric control valves, the whole machine, and large self-packaged valves	10,000 accumulated packaging tasks; meet the production of 10,0000 sets of water treatment valves per month	**3**
**3**	Installed two manipulators on the injection molding machine	Reduced the average injection molding cycle from 62 s to 55 s (11% increase in efficiency)	**2**
**4**	Purchased an integrated milling machine	Shortened the order delivery cycle by 20%; decreased related safety hazards	**1**
**5**	Achieved automatic stoppage by using control valves to compress nuts; attained automatic control over parker screws by linking them with positioning board and motors	Used one machine to perform the original two processes involving eight screws, reducing the working time by 22 s	**—**
**6**	Used ultrasonic processing technology to weld the installation slots of the base water distributor of the control valves to the slots that containing the center tube rubber	Realized automatic welding; reduced the processing unit time by 15 s; reduced occupational hazards	**1**
**7**	75 injection molding machines and blowing molding machines are equipped with waste gas collection devices which are collected on the roof and discharged after UV photo treatment	The workshop smell significantly improved; greatly reducing the physical harm of toxic gas to workers	—
**8**	The analysis of Man-machine operation and joint operation were carried out in the injection molding workshop, the free time of workers working in the workshop was explored, and the joint operation was improved	Improve the utilization rate of time, the workshop efficiency increased by about 3%	—
**9**	Reduce the number of existing products in the workshop, especially before and after the water test process	Solve a large number of products occupied evacuation channel affecting evacuation and passage	—
**10**	The torque test instrument display was redesigned to reduce it by 10cm	In line with the safety ergonomic engineering, reduce the waste of personnel action, to avoid the hidden danger caused by fatigue, the process work efficiency is increased by about 3% and 5%	—
**11**	Raise the height of the automatic packing line working table by 13cm	—	—
**12**	Online inkprinter is introduced in the product labeling link	The working efficiency is increased by 50%, and the workplace area is reduced by 45%	**2**
**13**	Ultrasonic welding position to add 2 manipulator instead of manual take the workpiece	Process working efficiency is increased by 24%	**2**
**14**	Optimize the operation process, avoid repeated loading of turnover boxes and handling, introduce intelligent transportation line, and introduce extended flexible conveyor belt	The extended flexible conveyor belt and automatic packaging transportation rail lines are optimized, which reduces waste, improves the working efficiency by about 14%	—
**15**	Control valve precision dry type sealing test equipment online trial	After repeated comparison and verification, the equipment has high accuracy and repeatability can realize automatic online detection.	—
**Total**			13

The above transformation measures enabled the enterprise to terminate seven production employees in the first year of implementation, 2 production employees in the second year, 4 production employees in the fourth year; remove two position with occupational hazards, significantly improve the efficiency of numerous production processes. Overall, the enterprise has promoted flexible just-in-time production, reduced capital turnover, and enhanced its overall profit.

### 5.3 Practical application results and effectiveness of SLSM mode

**Fig 9:**
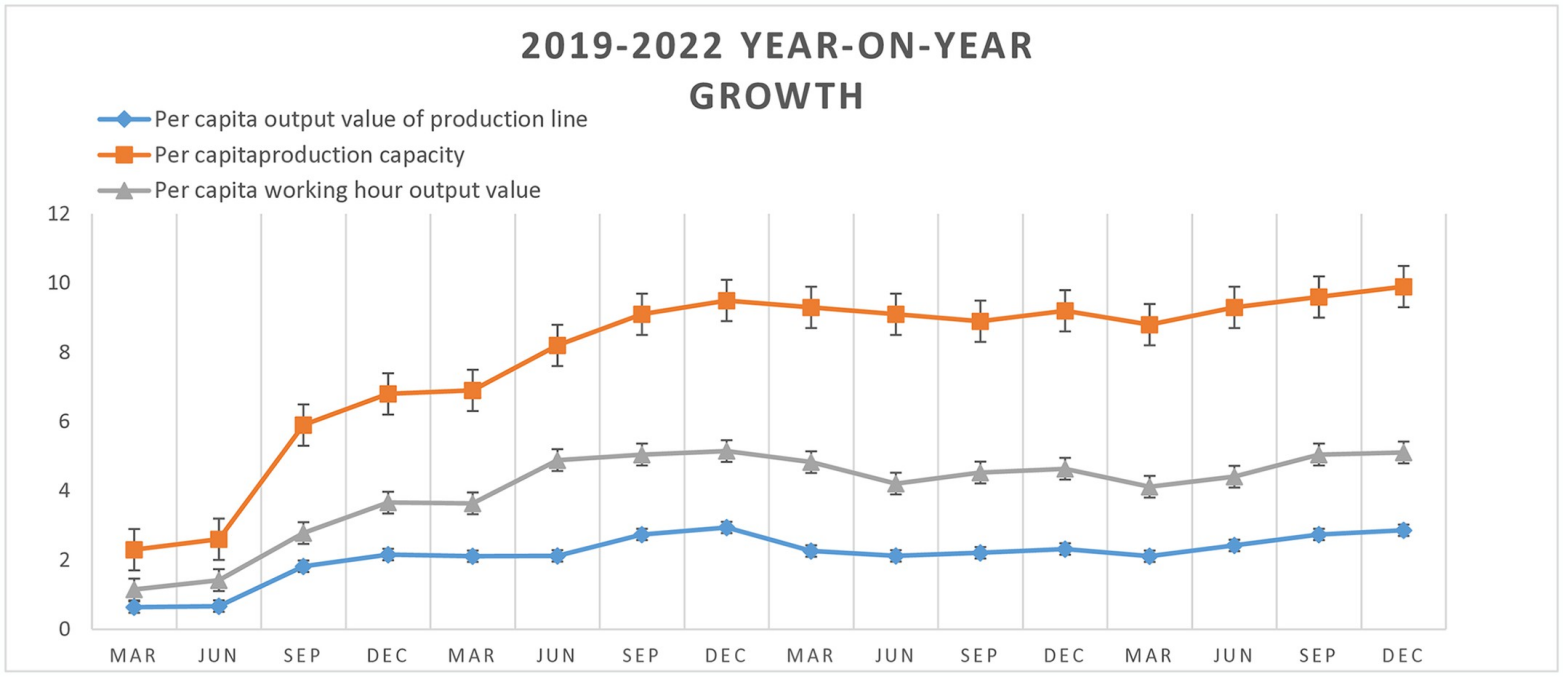
Year-on-year growth after SLSM mode implementation by Wenzhou-based machinery enterprise.

The above-mentioned transformation measures focus on industrial engineering, quality management and automatic grid control, and comprehensive management. The enterprise has revised its safety production regulations, enhanced related-party management systems, optimized specialized equipment and operations, and improved occupational health using dedicated software. A double-prevention mechanism was established, involving the classification of safety risks and the identification and elimination of hidden hazards, resulting in the identification of 187 risk points. Control measures were implemented for these risks, with control signs and responsible personnel assigned to manage 35 significant risks. Through the implementation of SLSM, the production line output per capita of 2019 increased by 2.16% from that in the previous year; furthermore, the production capacity per capita and production output per man-hour rose by 6.8% and 3.66% year-on-year, respectively. The production line output per capita of 2020 increased by 2.94% from that in the previous year; furthermore, the production capacity per capita and production output per man-hour rose by 9.5% and 5.15% year-on-year. (Owing to the COVID-19 pandemic, production operations were halted throughout the first quarter and the initial part of the second quarter of 2020. The computation of this data series incorporated scientific forecasts and assumptions, predicated on the production capabilities and outputs recorded in the fourth quarter of the preceding year and the second quarter of the current year. This methodology was implemented to more accurately reflect actual conditions, thus facilitating the completion of a closed-loop in the statistical process.) In 2021, per capita metrics in production line output, production capacity, and hourly production output witnessed increases of 2.32%, 8.9%, and 4.64%, respectively, compared to the end of the preceding year. In 2022, the data of production line output per capita, the production capacity per capita and the production output per man-hour were increased 5.11%, 9.9% and 2.86% respectively. The year-on-year growth after SLSM adoption was substantial compared with the previous year, as shown by the quarter on quarter growth curves in Fig 9.

## 6 Conclusion

1. In China, existing systematic safety management frameworks, legislation, and regulations are predominantly designed with medium and large enterprises in mind, often neglecting the unique needs of small and medium-sized enterprises (SMEs) in establishing a coherent safety management system. Consequently, there is a critical need to develop a contemporary safety management model and system that specifically addresses the sustainable development of SMEs.
2. Safety management constitutes an integral component of the organized production activities within SMEs. It is seamlessly integrated and complements the production and operational activities of SMEs. Scientific safety management contributes to the benign growth of economic benefits. Given the intense market competition across various industries, SMEs striving for survival and sustainable development should adopt an adaptive, scientifically-grounded safety management model.
3. The SQCDP management framework, employed to establish the proposed Safety and Lean Safety Management (SLSM) model for China’s SMEs, is structured into modules focusing on lean safety, quality, cost, and personnel. The implementation of the lean safety module involves one standardization, five improvements, and two new introductions. To realize the lean quality module, four processes and a comprehensive closed loop are proposed. The lean cost module is achieved through the strategic deployment of capital chains, detailed assessment of production factors, and effective reduction of personnel and time costs. The lean people management module is realized by enhancing allocation, training, attendance monitoring, inspection, and evaluation processes. The SLSM model is distinguished by its excellent self-correction capabilities both within and external to production systems, a beneficial closed loop among modules, a pronounced focus on enhancing corporate efficiency, and the capacity to maximize enterprise profits.窗体顶端
4. Wenzhou Runxin Machinery Enterprise successfully implemented the SLSM mode by integrating safety considerations into its personnel reduction strategies through automation, while also leveraging expert advice and support. Subsequently, the adoption of the revised safety management system in 2019 resulted in increases of 4.16% in production line output per capita, 14.5% in production capacity per capita, and 9.45% in production output per man-hour, compared to 2018 figures. In 2020, these metrics further demonstrated growth, with production line output per capita increasing by 2.94%, production capacity per capita by 9.5%, and production output per man-hour by 5.15%, all compared to the previous year’s end. The 2021 metrics for production line output per capita, production capacity per capita, and production output per man-hour are not considered to be of reference value. For 2022, relative to the previous year’s data, there was a 3.16% increase in the per capita output of production lines, a 6.5% rise in per capita production capacity, and a 2.91% enhancement in per capita output per working hour.
5. During the implementation of the SLSM mode, several key departments were established, including the R&D center, testing center, type laboratory, water quality testing room, and measurement room. Furthermore, guided by the SLSM mode, the company streamlined its workforce by 12 personnel, identified 142 risk points, and implemented corresponding controls. Additionally, automation transformations under this mode, along with the development of a collaborative platform involving government, industry, universities, and research institutions, have significantly enhanced the enterprise’s overall profitability and production efficiency.

## Supporting information

S1 Data (DOCX)
