# Supplementary material for: Development and effectiveness analysis of a safety management model for SMEs integrating lean management innovation with SQCDP framework
Source: PLoS One. 2025 Jan 13;20(1):e0316299. doi: 10.1371/journal.pone.0316299 (PMC11730395; doi:10.1371/journal.pone.0316299)
Supplement: S1 Data — (DOCX) [file pone.0316299.s001.docx]

The COVID-19 pandemic severely disrupted 2020 production, causing operations to cease during the first quarter and early second quarter. Scientific predictions and assumptions, based on the production capacity and output from the previous year's fourth quarter and the second quarter of the current year, were utilized to compute the relevant data. This methodology provided a more accurate representation of reality and enabled the statistical process to complete a closed loop. As a result, the total actual output and capacity for 2020 decreased. Despite these reductions, the application of these scientific and reasonable assumptions led to increases in calculated per capita production value, capacity, and production hours. Given the confidentiality constraints of detailed enterprise data, our team exerted considerable effort to maximize accuracy within the bounds of the available raw data. In response to the previously highlighted issue regarding the missing minimal dataset, we engaged in extensive communications over nearly two months. Regrettably, due to commercial confidentiality, we were unable to access the original data discussed in the paper. We deeply regret this situation and earnestly hope that the paper can still be published in your esteemed journal.

| 2019 | Total production value(In ten thousand yuan) | 47158 |
| --- | --- | --- |
|  | Total production capacity (In ten thousand units) | 47.08 |
|  | Per capita output value of production line (In ten thousand yuan per person) | 97.8 |
|  | Per capita production capacity (in ten thousand units per person) | 0.0976 |
| 2020 | Total production value (actual output) | 43477（after prediction：48545） |
|  | Total production capacity (actual capacity) | 46（after prediction：51.55） |
|  | Per capita output value of production line | 100.7 |
|  | Per capita production capacity | 0.107 |
| 2021 | Total production value | 49646 |
|  | Total production capacity | 55.912 |
|  | Per capita output value of production line | 103 |
|  | Per capita production capacity | 0.116 |
| 2022 | Total production value | 51092 |
|  | Total production capacity | 61.696 |
|  | Per capita output value of production line | 106 |
|  | Per capita production capacity | 0.128 |
